# Pregnancy and Delivery in a Woman With Achondroplasia: A Multidisciplinary Management Approach

**DOI:** 10.7759/cureus.98733

**Published:** 2025-12-08

**Authors:** Falak Baloch, Liliana Grosu

**Affiliations:** 1 Department of Obstetrics and Gynaecology, Bedfordshire Hospitals NHS Foundation Trust, Bedford, GBR

**Keywords:** achondroplasia, achondroplastic, combined spinal epidural, dwarfism, pregnancy

## Abstract

Achondroplasia is the most common skeletal dysplasia, characterised by disproportionate short stature and associated with obstetric and anaesthetic challenges, with limited evidence on optimal pregnancy management. We report a 39-year-old Gravida 3 Para 2 woman with achondroplasia and two previous caesarean sections who received consultant-led, multidisciplinary antenatal care involving obstetrics, anesthesiology, cardiology, and neonatology teams. Her pregnancy was complicated by short stature, prior caesarean deliveries, and previous children affected by achondroplasia. She underwent an elective lower segment caesarean section and bilateral tubal ligation at 37 weeks under combined spinal-epidural anesthesia, delivering a live female infant weighing 2,760 grams (94.6th centile) with Apgar scores of 9 and 10. Postoperative maternal and neonatal recovery was uneventful, and the neonate showed no clinical, radiological, or genetic evidence of achondroplasia. A tailored multidisciplinary approach, early anesthetic assessment, careful delivery planning, prenatal counseling, and risk evaluation, including discussion of invasive prenatal testing, were crucial to minimize maternal and neonatal complications. Maternal postoperative care included monitoring for hypertensive episodes and adjustment of obstetric hemorrhage thresholds due to body habitus.

This case highlights that pregnancy in women with achondroplasia requires individualized, multidisciplinary care to optimize maternal and neonatal outcomes, with early consultation, detailed anomaly scanning, anesthetic planning, and comprehensive risk assessment being key to successful management.

## Introduction

Achondroplasia is the most common form of skeletal dysplasia, characterised by disproportionate short stature due to rhizomelic limb shortening and distinctive craniofacial features [1]. Its prevalence is estimated at 3.72 per 100,000 live births [2]. The condition is caused by a pathogenic variant in the Fibroblast Growth Factor Receptor 3 (FGFR3) gene, which disrupts endochondral ossification during skeletal development [3]. Individuals with achondroplasia may face lifelong complications, including spinal abnormalities, restrictive lung disease, and early childhood mortality [4].

Pregnancy in women with achondroplasia is rare and associated with significant obstetric and anaesthetic challenges. Women with this condition are at increased risk of infertility, menorrhagia, dysmenorrhea, leiomyomata, and early menopause [4-6]. Obstetric complications include cephalopelvic disproportion (CPD) necessitating caesarean delivery (CS), preterm birth (PTB), and fetal skeletal dysplasia. At the same time, maternal cardiopulmonary risks are also elevated due to restrictive lung disease and potential cardiac anomalies such as congenital heart diseases, cardiomyopathy, coronary artery disease, and pulmonary hypertension [6-8].

Despite concerns about skeletal abnormalities complicating epidural anaesthesia, clinical experience shows no serious complications. While technical issues like dural puncture have occurred, subsequent attempts have been successful. The literature on anaesthetic management for caesarean sections in achondroplasia patients is reviewed, focusing on local anaesthetic choice, epidural test doses, and dose titration [9]. Currently, no clear guidelines exist for anaesthetic management in pregnancy; decisions should be made on a case-by-case basis following a thorough assessment of risks and benefits [10,11].

Women with achondroplasia may face reduced fertility due to pelvic and hormonal factors and often require lifestyle adaptations to accommodate skeletal limitations during pregnancy. Current international consensus and clinical guidelines recommend a multidisciplinary approach, including early anaesthetic assessment, detailed anomaly scans, individualised delivery planning, and counselling regarding potential maternal and fetal complications [12,13].

Given the limited literature on management strategies and outcomes, reporting individual cases is essential to guide multidisciplinary planning and safe delivery. This case highlights the antenatal, anaesthetic, and obstetric management of a pregnant woman with achondroplasia delivering a healthy neonate.

## Case presentation

A 39-year-old Gravida 3 Para 2 (G3P2) woman with achondroplasia was booked under consultant-led care at 13 weeks of gestation. The patient had a known history of bilateral hearing impairment and required the use of hearing aids for daily communication. She had a history of two previous caesarean sections and two children with achondroplasia. Her height was 147 centimetres (cm), weight 31 kilograms (kg), giving a Body Mass Index (BMI) of 22.65 kilograms per square meter (kg/m^2^). She was allergic to penicillin, which remained unresolved, and penicillin was not administered during her care. She was a known smoker, with a history of 10 cigarettes per day and a carbon monoxide level of 12 ppm at booking. Her obstetric history included prior small-for-gestational-age (SGA) infants delivered by caesarean section due to breech presentation. Key maternal and fetal characteristics, obstetric history, antenatal complications, multidisciplinary care, anaesthetic plan, and delivery outcomes are summarised in Table 1.

**Table 1:** Maternal and fetal characteristics and outcomes This table summarises the key maternal demographics, obstetric history, antenatal complications, multidisciplinary care, anaesthetic plan, and delivery outcomes for a 39-year-old woman with achondroplasia. Neonatal outcomes, including birth weight, Apgar scores, and absence of achondroplasia, are also included.

Parameter	Details
Maternal Age	39 years
Gravida / Para	Gravida 3 Para 2
Height	147 cm
Weight / BMI	31 kg / 22.65 kg/m²
Medical History	Achondroplasia, previous caesarean sections, past SGA infants, smoker (reduced but on and off smoking)
Allergies	Penicillin
Antenatal Complications	Short stature, risk of cephalopelvic disproportion, prior achondroplasia in offspring
Multidisciplinary Care	Obstetrics, anaesthesiology, cardiology, neonatology
Anaesthetic Plan	Combined spinal-epidural anaesthesia
Delivery	Elective lower segment caesarean section with bilateral tubal ligation at 37 weeks
Neonatal Outcome	Female infant, 2,760 g (94.6th centile), Apgar 9/10, unaffected by achondroplasia
Maternal Outcome	Uneventful postoperative course, BP monitored, Venous Thromboembolism risk assessed and actioned

The patient received multidisciplinary (MDT) care throughout pregnancy, involving obstetrics, anaesthesiology, cardiology, and neonatology teams. The plan by the obstetric team at the booking visit was to start her on aspirin 150 mg till 36 weeks of pregnancy, keeping in view her history of a previous small for gestational age baby. She was booked with NHS quit smoking services, which she initially declined; however, upon explaining how it could affect the baby, she accepted the referral. She was informed of the implications of SGA, FGR, preterm labour, and placental abruption as a consequence of smoking. She was booked for an anomaly scan and uterine artery Doppler at 20 weeks’ gestation. Further growth scans were planned based on the Doppler results. If the uterine artery Dopplers were abnormal, serial growth scans were to commence at 28 weeks. If normal, growth scans were scheduled for 32, 36, and 39 weeks of gestation. A plan was made to deliver her via an elective caesarean section at 39 weeks, subject to review if complications arose. An anaesthetic referral was made to formulate an intrapartum anaesthesia plan, with particular attention to determining the threshold for major obstetric haemorrhage in view of her body habitus. Blood pressure and urine monitoring were planned every 4 weeks until 28 weeks’ gestation, every 2 weeks until 36 weeks, and weekly thereafter, as recommended for all antenatal women. She received routine antenatal vaccinations and was provided with smoking-cessation support.

She underwent an anomaly scan with uterine artery Doppler at 20+2 weeks of gestation. Both upper and lower limbs appeared normal for the gestation, and the rest of the anomaly scan was normal with no abnormalities detected; however, there was a limited view of the spine in the coronal section. Left uterine artery Doppler traces were found to be abnormal with evidence of notching and a mean pulsatility Index of 1.4. Therefore, a referral was made to the Fetal Medicine unit (FMU) for further assessment and planning. She was seen by the FMU at 21 weeks of pregnancy and underwent a repeat anomaly scan, which demonstrated normal findings and appropriate fetal growth for gestational age, with no obvious structural abnormalities.

She was counselled regarding the risks of genetic inheritance of achondroplasia, and amniocentesis was discussed with a clear explanation of the approximately 1% procedure-related miscarriage risk. She declined invasive testing, as she was keen to continue with the pregnancy even if the fetus was affected. She was informed that it is not possible to detect all abnormalities with ultrasound scans, and chromosomal or genetic syndromes cannot be confirmed or excluded by ultrasound scans alone.

Her subsequent ultrasound scan at 29 weeks of pregnancy showed an estimated fetal weight on the 99th centile, with normal liquor volume and umbilical artery Dopplers. In view of the fetal size, a referral was made to the diabetic team for an oral glucose tolerance test (OGTT). She was counselled on the risks associated with a large-for-gestational-age (LGA) fetus, including postpartum haemorrhage. She was also informed that shoulder dystocia and perineal trauma were not anticipated risks in her case, as she was already planned for an elective caesarean section.

She did not attend her initial OGTT appointment and was rebooked, which she again did not attend. A third appointment was arranged; however, she smoked prior to attendance. She was subsequently advised to perform seven-day capillary blood glucose monitoring, which she attempted but did not complete. This led to diagnostic challenges, as there was inadequate evidence to confirm a diagnosis of gestational diabetes mellitus.

She underwent another growth scan at 33 weeks of gestation, which was suggestive of accelerated growth velocity, with an estimated fetal weight above the 97th centile, polyhydramnios (AFI 26.4 cm, above the normal range), and newly identified fetal ascites. Another referral was made to the FMU, and TORCH (Toxoplasma, Cytomegalovirus, Rubella, Herpes, and Parvovirus) screening was arranged. A paediatric alert was also raised.

She was seen by the FMU again at 33+6 weeks in view of the polyhydramnios and fetal ascites. The repeat scan showed minimal fetal ascites, with no pleural or pericardial effusion and no evidence of hydrops. The middle cerebral artery Doppler was < 1.5 multiples of median (MoM), with no signs of fetal anaemia or tricuspid regurgitation. The ductus venosus Doppler was normal. She was counselled regarding the findings of minimal fetal ascites with no evidence of anaemia or hydrops and informed that this could be related to fetal infection (TORCH results were still pending).

The plan was to repeat the scan locally in one week and to proceed with a planned caesarean section at 37 weeks, followed by postnatal assessment of the baby. Maternal cardiac echocardiography (to assess cardiac anatomy and rule out pulmonary hypertension) was recommended by the FMU, as she reported breathlessness; however, her oxygen saturation remained normal. The neonatology team planned to assess the baby at delivery and determine the need for further investigations.

At 35+6 weeks, she was referred to the delivery suite by her general practitioner (GP) with complaints of vomiting and abdominal pain. Her urine dipstick was positive for nitrites, and she was started on cephalexin for a suspected urinary tract infection; a urine sample was sent for culture and sensitivity. She was experiencing mild palpable uterine contractions with mild scar tenderness. A speculum examination showed that the cervical os was closed, and she was admitted for observation.

She underwent a repeat ultrasound scan locally at 36 weeks to assess for liquor volume and Dopplers. The findings were unchanged from the previous scan, with minimal fetal ascites and normal umbilical artery dopplers. Following the scan, a plan was made to reschedule the caesarean section for 37 weeks (as advised by the FMU) and to discharge her from the hospital.

She underwent an anaesthetic assessment at 36 weeks prior to her caesarean section, and the plan was to crossmatch two units of packed red cells (PRC), proceed with a combined spinal-epidural (CSE), and have a consultant anaesthetist where possible. The planned spinal dose was 1 mL of heavy Marcaine without opioids. Postoperative medications included paracetamol 450 mg four times a day, ibuprofen 200 mg four times a day and oral morphine 6-9 mg every four hours for postoperative pain management.

Antenatal investigations and fetal growth assessments, including anomaly scans, echocardiography, and blood tests, are summarised in Table 2.

**Table 2:** Antenatal investigations and monitoring This table summarises the key antenatal investigations, imaging, and maternal monitoring for a 39-year-old woman with achondroplasia, including fetal growth assessments, anomaly scans, maternal echocardiography, and relevant laboratory results during pregnancy. FMU: Fetal Medicine unit; TORCH: Toxoplasma, Cytomegalovirus, Rubella, Herpes, and Parvovirus; MRSA: Methicillin-resistant Staphylococcus aureus

Investigation / Scan	Result	Gestational Age
Anomaly Scan	Normal fetal anatomy, no evidence of achondroplasia; left uterine artery notching	20+2 weeks
FMU Scan	Normal fetal anatomy; appropriately grown fetus	21+0 weeks
Growth Scan	Estimated fetal weight (EFW) > 99th centile; fetal presentation: cephalic; normal Liqour volume and Dopplers	29+0 weeks
Growth Scan	Fetal ascites; polyhydramnios (AFI:26.4 cm); EFW > 97th centile	33+0 weeks
FMU Scan	AFI: 13.9 cm, MCA PSV <1.5 MoM; minimal fetal ascites; no pleural or pericardial effusion	33+6 weeks
FMU Scan	Minimal fetal ascites; no pleural or pericardial effusion	35+1 weeks
Liquor Volume and Doppler Scan	Normal Dopplers and Liquor Volume	36+0 weeks
Echocardiography (Maternal)	No structural abnormality; good RV and LV function; ejection fraction 55%	36+ weeks
Maternal Blood Tests	Normal haemoglobin, platelets, and urine	Booking
Blood Tests	Haemoglobin 100 g/L	28+0 weeks
TORCH Screen	Negative	33+0 weeks
Blood Tests	Haemoglobin 94 g/L	33+0 weeks
MRSA Swabs	Not detected	36+0 weeks
Group and Save	No atypical antibodies detected	Booking, 28 weeks, at delivery

At 37 weeks, an elective lower segment caesarean section (transverse uterine incision) with bilateral tubal ligation, performed under combined spinal-epidural (CSE) anaesthesia, was carried out due to cephalopelvic disproportion and two prior caesarean deliveries. Intraoperatively, dense adhesions were noted in the lower uterine segment. A healthy female infant weighing 2,760 grams (94.6th centile) was delivered with Apgar scores of 9 and 10. Estimated blood loss (EBL) during the surgery was 150 ml, and postoperative recovery was uneventful, with both mother and neonate discharged after three days. Postoperative haemoglobin was 87 g/L, and she was commenced on oral ferrous sulphate 200 mg daily. The neonate had no clinical or radiological evidence of achondroplasia. She was discharged with 6 weeks of Tinzaparin, prescribed at 2500 international units based on her booking weight, for venous thromboprophylaxis.

## Discussion

Achondroplasia is the most common skeletal dysplasia, characterised by disproportionate short stature due to rhizomelic limb shortening and craniofacial features [1]. It arises from a pathogenic variant in the FGFR3 gene, disrupting endochondral ossification during fetal development [3]. Although relatively rare, pregnancy in women with achondroplasia poses significant obstetric and anaesthetic challenges due to maternal skeletal and cardiopulmonary limitations, as well as potential fetal complications such as hydrocephalus, thoracic cage abnormalities, and fetal demise [2,4,11].

Women with achondroplasia have increased risks of infertility, menorrhagia, dysmenorrhea, leiomyomata, and early menopause, which may limit reproductive potential and complicate antenatal care [5,6]. Obstetric risks include cephalopelvic disproportion, preterm birth, and higher rates of caesarean delivery [3,8]. Maternal cardiopulmonary compromise is common due to a small thoracic cavity, restrictive lung disease, scoliosis, and increased abdominal pressure during pregnancy, which can exacerbate dyspnea and reduce functional residual capacity [3,7,8].

Caesarean delivery is generally recommended due to the high likelihood of cephalopelvic disproportion and to avoid risks associated with prolonged labour, including spinal cord compression in the fetus due to restricted foramen magnum and cervical spine compression [8]. Anaesthetic management is challenging; both regional and general anaesthesia may be technically difficult. Regional anaesthesia is preferred when feasible, although dense spinal anatomy and altered landmarks can complicate catheter placement [8-10]. Careful preoperative anaesthetic assessment and planning are essential, as demonstrated in this case, where combined spinal-epidural anaesthesia was successfully administered.

Prenatal care should include detailed anomaly scanning by fetomaternal specialists and consideration of invasive prenatal testing for achondroplasia, particularly if one or both parents are affected [6]. Genetic counselling is important to inform parents about inheritance patterns and the 50% risk of achondroplasia when an affected parent has a partner of average stature [6].

This case demonstrates that with multidisciplinary, individualised planning and favourable maternal and neonatal outcomes are achievable. Early anaesthetic assessment, consultant-led antenatal care, and coordination with neonatology are crucial for safe delivery. While the neonate in this case was unaffected, monitoring for potential complications and long-term follow-up remain important.

## Conclusions

Pregnancy in women with achondroplasia is high-risk and requires a multidisciplinary, individualised approach to optimise maternal and neonatal outcomes. Consultant-led care with regular antenatal monitoring is essential, along with the involvement of anaesthesiologists, pulmonologists, cardiologists, neonatologists, and specialist midwives. Detailed anomaly scanning and consideration of invasive prenatal testing should be offered when indicated. Caesarean delivery is often necessary due to cephalopelvic disproportion, and perioperative planning should account for maternal body habitus, including adjusted thresholds for obstetric haemorrhage. Early anaesthetic assessment ensures the safe administration of regional or general anaesthesia. This case demonstrates that proactive risk assessment and collaborative planning can lead to favourable outcomes for both mother and baby.
